# A versatile automatic gas-sampling system

**DOI:** 10.1155/S1463924683000255

**Published:** 1983

**Authors:** H. L. Reed, D. W. Oliver, A. Tavener

**Affiliations:** The Meat Industry Research Institute of New Zealand (Inc.) P.O. Box, 617 Hamilton New Zealand


Journal of Automatic Chemistry, Volume 5, Number 2 (April–June 1983), pages 94–98

### A versatile automatic gas-sampling system

**H. L. Reed, D. W. Oliver and A. Tavener**
*The Meat Industry Research Institute of New Zealand (Inc.), P.O. Box, 617, Hamilton, New Zealand*

### Introduction

Commercially available equipment for automatic gas-sampling is both expensive and bulky. Air infiltration into refrigerated areas should ideally be measured with low-cost, portable, automatic gas-samplers, capable of operating in a wide range of ambient temperatures. This paper describes such a sampler. Although the need for portability limits the size of the equipment and therefore the volume of sampled gas, the consequent reduction in accuracy was not significant in this application. Depending on which tracer gas is used, its concentration in the sampled gas can be determined by infra-red spectroscopy or gas chromatography.

The portable gas-sampler was used to determine the infiltration of warm air into a number of single-storey cold-rooms with volumes from 4000 to 38 000 $m^3$ and various types of door protection [1]. Infiltration was determined by measuring the rate of decay of the tracer gas $SF_6$ (sulphur hexafluoride). If the tracer gas becomes well mixed with the air in the room, the rate at which its concentration, $c$, decreases with time can be related to the rate of air change, $r$ (i.e. the infiltration rate divided by the volume of the room) by:

$$\ln(c/c_0) = -rt$$

where $c_0$ is the initial concentration and $t$ is the time after injection.

Sulphur hexafluoride can be detected by electron-capture gas chromatography at concentrations down to one part in $10^{13}$ [2]. It is completely harmless to people and products, relatively inexpensive, and thus ideal for tests in large rooms, such as meat cold-stores.

### Description

The sampler mechanism consists of a rotating drum, or turntable, housing a number of vertically positioned, vacuum-sealed, 20 ml test-tubes. The turntable is mounted on a vertical axle, which is rotated in steps by a modified uniselector mechanism to position each sampling tube directly beneath a hypodermic needle (figure 1). For measurements of air infiltration into cold-stores 24 test-tubes are used. The hypodermic needle is connected via a flexible tube to a small diaphragm pump, which in turn is connected via tubing to the area from which the gas is to be sampled. A reciprocating mechanism, driven by a pulsed d.c. motor, moves the hypodermic needle downward to penetrate the rubber bung of the sample tube, thereby exposing it to the test atmosphere (figure 2). After a pre-set delay the needle is withdrawn upward from the self-sealing bung. The turntable is rotated in two steps to position the next sampling tube beneath the needle, and an LED starts flashing at 1 s intervals to indicate that timing is taking place.

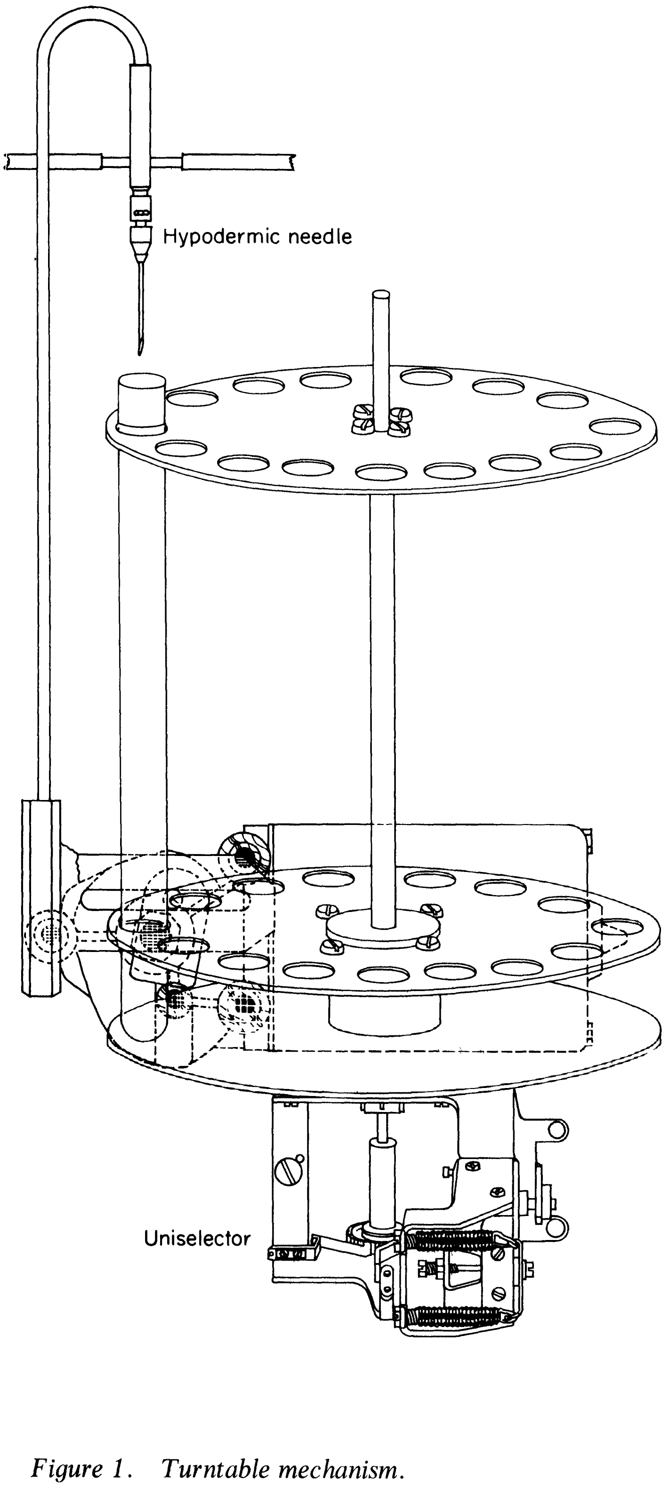

*Figure 1. Turntable mechanism.*

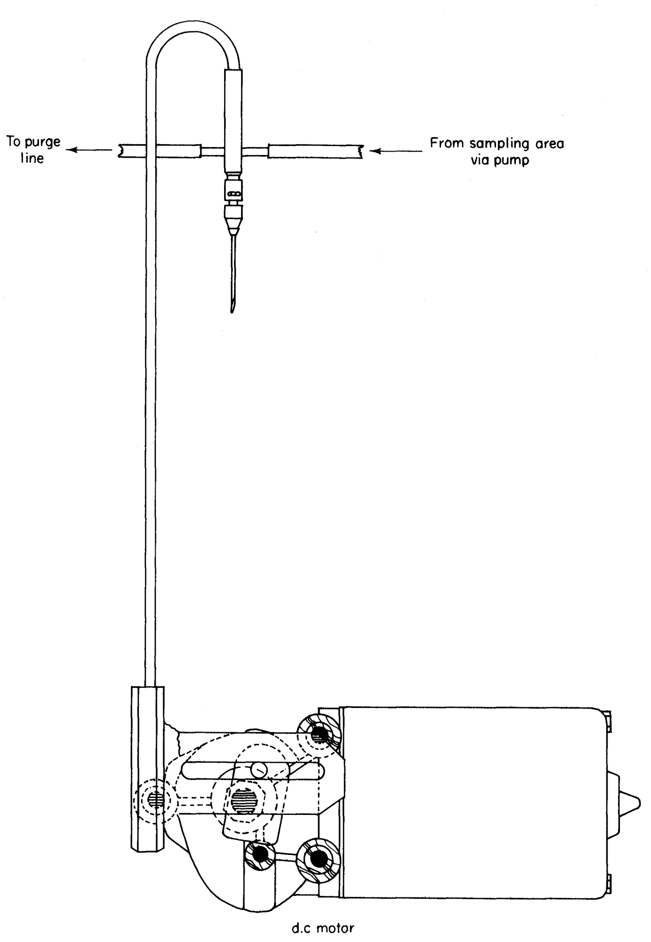

*Figure 2. Needle mechanism.*

The timing interval is determined by the setting of a decade thumbwheel switch, each digit representing 10 min. Because the thumbwheel switch setting is 'read' at the start of each sampling interval, the interval can be changed during sampling, if required, by changing the switch setting. The sampler will automatically fill all the sample tubes and then stop, regardless of the time interval setting.

The microprocessor not only controls the timing intervals and stepping sequences, it also regulates the torque of the d.c. motor to reduce 'over run' after switch-off. A stand-by battery provides immunity against short-term power failures. A flow diagram is shown in figure 3 and an assembly listing is given in figure 4. Full documentation of the system is available from the authors.

There are six push-button control switches. The 'Auto' button starts the automatic sampling sequence; 'Start test', 'Stop test', 'Reset', 'Motor' and 'Step' buttons allow the operator to check that the automatic control system and mechanism are functioning correctly before the machine is operated in the automatic mode.

*Start test*
The Start test button is used to check that the mechanism is functioning correctly. In this mode the motor moves the needle down until it operates a microswitch at the bottom of its stroke. After a short pause the needle is moved up until it operates a microswitch at the top of its stroke. At this point the motor stops and the uniselector is stepped twice to position the next sample tube under the needle, after which the cycle is repeated. This 'fast test cycle' function runs continuously until the Stop test button is pressed.

*Stop test*
Operation of the Stop test button terminates the 'fast test cycle' at the end of a complete test cycle, at which point an LED glows to indicate that the mechanism and control system are ready for automatic sampling.

*Reset*
The Reset button can be used at any point in the sampling sequence to terminate the operation and restore the system to manual control.

*Motor*
When the Motor button is depressed, the motor actuating the needle movement turns slowly until the button is released.

*Step*
When the step button is depressed, the uniselector steps continuously to rotate the turntable.

*Auto*
The Auto button is depressed to start the actual sampling sequence. In this mode the Start test, Stop test, Motor and Step buttons are disabled.

### Construction

The sampler is housed in two metal electrical-distribution cabinets (457 x 305 x 203 mm), each having a hinged door. The cabinets are mounted back-to-back, one containing the electronics and the diaphragm pump, the other containing the sampling tubes and the associated mechanism. The turntable can be tilted forward to enable the sampling tubes to be inserted or removed. Provision is made for the internal stowage of the mains-power lead. The cabinet housing the electronics is partially lined with 25 mm sheet polystyrene to retain some of the locally generated heat. The total weight of the equipment is 25·5 kg.

### Applications

The device can be used for any application in which a gas is sampled to measure its concentration. The only service required is a mains-power supply. Minor circuit changes would enable the sampler to be battery powered. A simple modification to the rotating drum will cater for a different size of test-tube or vial. Commerical pre-evacuated tubes can provide a sampled gas volume accuracy of $\pm 1\%$ [1]. The sampler can also be used to pre-evacuate sampling tubes by connecting the hypodermic needle to a vacuum pump. The use of a side-entry syringe would allow the device to sample liquids from continuous processes or batch reactions. The physical size, versatility and low cost of the sampler should enable its use as a multi-purpose laboratory apparatus.

### References

1. Pham, Q. T. Air infiltration into cold stores. In *Proceedings of 22nd Meat Industry Research Conference* (Hamilton, New Zealand, 1982).
2. Grimsrud, D. T., Sherman, M. H., Janssen, J. E., Pearman, A. N. and Harrje, D. T. *ASHRAE Transactions*, **86**, 1 (1980), 285.

*Figures 3 and 4 follow.*

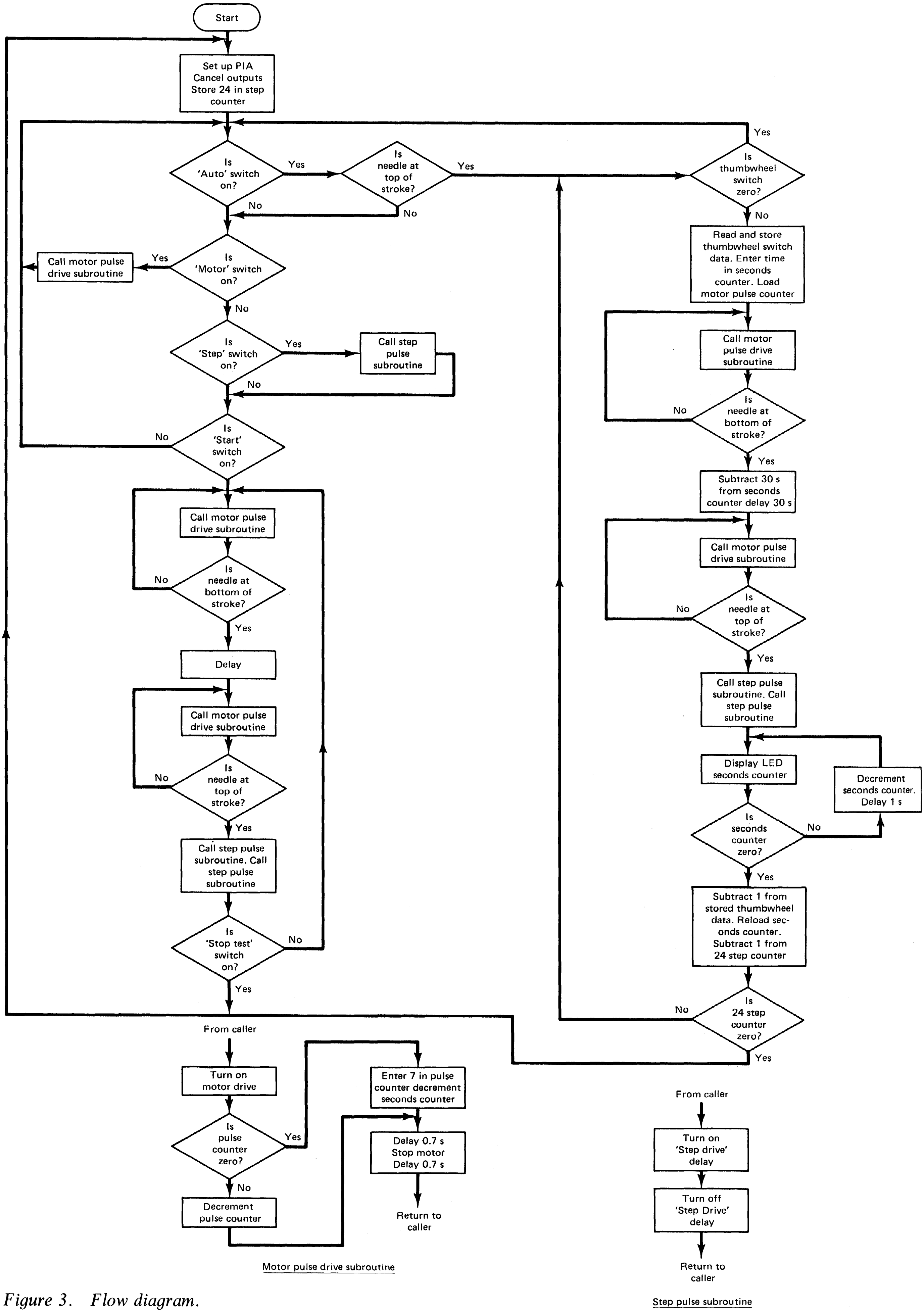

*Figure 3. Flow diagram.*

```

6802 MICRO ASSEMBLER/DISASSEMBLER

1000: $CO

1000—  8E   LDS        $007F    SET UP P.I.A.s

1003—  CE   LDX        $8000    CLEAR OUTPUTS

1006—  DF   STX         10

1008—  86   LDA    A   $FF

100A—  A7   STA    A    00, X

100C—  A7   STA    A    04, X

100E—  86   LDA    A   $00

1010—  A7   STA    A    02, X

1012—  A7   STA    A    06, X

1014—  C6   LDA    B   $04

1016—  E7   STA    B    01, X

1018—  E7   STA    B    03, X

101A—  E7   STA    B   05, X

101C   E7   STA    B    07, X

101E—  6F   CLR         00, X

1020—  86   LDA    A   $18      LOAD 24 SAMPLE COUNTER

1022—  97   STA    A   20

1024—  A6   LDA    A    02, X   TEST AUTO PUSH BUTTON

1026—  84   AND    A   $01

1028—  26   BNE         1048

102A—  A6   LDA    A    02, X   TEST MOTOR PUSH BUTTON

102C—  84   AND    A   $08

102E—  27   BEQ         1035

1030—  BD   JSR         F120

1033—  20   BRA         1024

1035—  A6   LDA    A    02, X   TEST STEP PUSH BUTTON

1037—  84   AND    A   $10

1039—  27   BEQ         1040

103B—  BD   JSR         F0F0

103E—  20   BRA         1035

1040—  A6   LDA    A    02, X   TEST START TEST PUSH BUTTON

1042—  84   AND    A   $40

1044—  26   BNE         10C2

1046—  20   BRA         1024

1048—  A6   LDA    A    02, X         (START OF AUTOMATIC CYCLE)

104A—  84   AND    A   $02      TEST NEEDLE UP

104C—  27   BEQ         102A

104E—  A6   LDA    A    06, X   READ THUMBWHEEL SWITCH

1050—  84   AND    A   $0F      STORE 10 MINUTE INTERVAL COUNTER

1052—  27   BEQ         1024

1054—  97   STA    A   17

1056—  86   LDA    A   $07      LOAD MOTOR PULSE COUNTER

1058—  97   STA    A   16

105A—  CE   LDX        $025C

105D—  DF   STX         18

105F—  BD   JSR         F120    CALL MOTOR PULSE DRIVE SUBROUTINE

1062—  A6   LDA    A    02, X   TEST NEEDLE DOWN

1064—  84   AND    A   $04

1066—  27   BEQ         105F    SUBTRACT 30 SECONDS FROM SECONDS COUNTER

1068—  0C   CLC

1069—  96   LDA    A   19

106B—  80   SUB    A   $1E

106D—  97   STA    A   19

106F   96   LDA    A   18

1071—  82   SBC    A   $00

1073—  97   STA    A   18

1075—  86   LDA    A   $38

1077—  CE   LDX        $FFFE    DELAY 30 SECONDS

107A—  09   DEX

107B—  26   BNE         107A

107D   4A   DEC    A

107E—  26   BNE         1077

1080—  BD   JSR         F120    CALL MOTOR PULSE DRIVE SUBROUTINE

1083—  A6   LDA    A    02, X   TEST NEEDLE UP

1085—  84   AND    A   $02

1087—  27   BEQ         1080

1089—  BD   JSR         F0F0    CALL STEP PULSE ROUTINE

108C—  BD   JSR         F0F0    CALL STEP PULSE SUBROUTINE

108F—  DE   LDX         10      PULSE AUTO LED

1091—  96   LDA    A    19

1093—  A7   STA    A    04, X

1095—  DE   LDX         18      TEST SECONDS COUNTER ZERO

1097—  26   BNE         10B1

1099—  D6   LDA    B    17      COUNT DOWN 10 MINUTE INTERVAL COUNTER

109B—  5A   DEC    B

109C—  D7   STA    B    17

109E—  27   BEQ         10A7

10A0—  CE   LDX        $025C    RELOAD SECONDS (10 MINUTE) COUNTER

10A3—  DF   STX         18

10A5—  20   BRA         108F

10A7—  DE   LDX    10

10A9—  7A   DEC         0020    COUNT DOWN 24 SAMPLE COUNTER

```

*Figure 4 (continued over).*

```

10AC—  26  BNE        104E

10AE—  7E  JMP        F000     LAST SAMPLE RETURN TO START

10B1—  CE  LDX        $F3E9    DELAY 1 SECOND COUNT DOWN 1 SECOND

10B4—  01  NOP                 REPEAT

10B5—  FF  STX        001A

10B8—  09  DEX

10B9—  26  BNE        10B4

10BB—  DE  LDX        18

10BD—  09  DEX

10BE—  DF  STX        18

10C0—  20  BRA        108F          (START OF TEST CYCLE)

10C2—  BD  JSR        F120     PULSE MOTOR

10C5—  A6  LDA   A    02, X    TEST NEEDLE DOWN

10C7—  84  AND   A    $04

10C9—  27  BEQ        10C2

10CB—  C6  LDA   B    $04      DELAY

10CD—  CE  LDX        $0000

10D0—  09  DEX

10D1—  26  BNE        10D0

10D3—  5A  DEC   B

10D4—  26  BNE        10CD

10D6—  BD  JSR        F120     CALL MOTOR PULSE SUBROUTINE

10D9—  A6  LDA   A    02, X    TEST NEEDLE UP

10DB—  84  AND   A    $02

10DD—  27  BEQ        10D6

10DF—  BD  JSR        F0F0     CALL STEP SUBROUTINE

10E2—  BD  JSR        F0F0     CALL STEP SUBROUTINE

10E5—  A6  LDA   A    02, X    TEST CANCEL BUTTON

10E7—  84  AND   A    $20

10E9—  27  BEQ        10C2

10EB—  7E  JMP        F000     RETURN TO START

10EE—  00  00

10EF—  00  00

10F0—  86  LDA   A    $02      TURN STEP PULSE ON        (STEP SUBROUTINE)

10F2—  A7  STA   A    00, X

10F4—  CE  LDX        $4000    DELAY

10F7—  BD  JSR        F110

10FA—  86  LDA   A    $00      TURN STEP PULSE OFF

10FC—  A7  STA   A    00, X

10FE—  CE  LDX        $4000    DELAY

1101—  BD  JSR        F110

1104—  DE  LDX        10

1106—  39  RTS                 RETURN TO CALLER

1107—  00  00

1108—  00  00

1109—  00  00

110A—  00  00

110B—  00  00

110C—  00  00

110D—  00  00

110E—  00  00

110F—  00  00

1110—  FF  STX        001A     DELAY

1113—  09  DEX

1114—  26  BNE        1110

1116—  DE  LDX        10

1118—  39  RTS

1120—  86  LDA   A    $01      OUTPUT MOTOR PULSE        (MOTOR DRIVE SUBROUTINE)

1122—  DE  LDX        10

1124—  A7  STA   A    00, X

1126—  96  LDA   A    16       TEST FOR END OF SECOND

1128—  26  BNE        1135

112A—  86  LDA   A    $07      START NEW SECOND

112C—  97  STA   A    16

112E—  DE  LDX        18       COUNT DOWN SECONDS COUNTER

1130—  09  DEX

1131—  DF  STX        18

1133—  20  BRA        1138

1135—  4A  DEC   A             COUNT DOWN END OF SECOND TIME

1136—  97  STA   A    16

1138—  CE  LDX        $0800

113B—  FF  STX        0012     DELAY FOR ON TIME

113E—  09  DEX

113F—  26  BNE        113B

1141—  86  LDA   A    $00      CANCEL MOTOR PULSE

1143—  DE  LDX        10

1145—  A7  STA   A    00, X

1147—  CE  LDX        $1AD0    DELAY FOR OFF TIME

114A—  FF  STX        0012

114D—  09  DEX

114E—  26  BNE        114A

1150—  DE  LDX        10

1152—  39  RTS                 RETURN TO CALLER

```

*Figure 4. Assembly listing.*

